# Deaths and hospitalizations of victims of non-drug toxicological events in Brazil, 2009 to 2018

**DOI:** 10.1590/S2237-96222022000300018

**Published:** 2022-12-19

**Authors:** Fernanda Gross Duarte, Sandra da Silva Moreira, Maria Goreth Barberino, Marcelo Neubauer de Paula, Nelzair Araújo Vianna, Maria Conceição Chagas de Almeida, Edson Duarte Moreira

**Affiliations:** 1 Instituto Gonçalo Moniz, Laboratório de Epidemiologia Molecular e Bioestatística, Salvador, BA, Brazil; 2 Associação Obras Sociais Irmã Dulce, Centro de Pesquisa Clínica, Salvador, BA, Brazil; 3 Grupo Hypera Pharma, São Paulo, SP, Brazil

**Keywords:** Intoxication, Mortality, Hospitalization, Toxicology, Time Series Studies

## Abstract

**Objective:**

to determine the rate of hospitalizations due to acute non-drug poisoning (NDP) events and to analyze mortality arising from these health conditions in Brazil from 2009 to 2018.

**Methods:**

this was a time-series study using Prais-Winsten regression to analyze records of hospitalizations for “treatment of intoxication or poisoning due to exposure to non-drug substances” held on the Hospital Information System.

**Results:**

there were 125,570 hospitalizations due to NDP. The average hospitalization rate was 6.3/100,000 inhabitants, although it was higher in males (8.0/100,000 inhab.) compared to females (4.6/100,000 inhab.). The hospitalization rate and the overall mortality rate due NDP to fell from 9.4 to 4.5/100,000 inhab. and from 2.5 to 1.6/1 million inhab., respectively.

**Conclusions:**

there was a reduction in the NDP hospitalization rate and in mortality due to NDP during the decade analyzed.

**Study contributions**

**Main results**

The hospitalization rate and the overall hospitalization mortality rate due to non-drug poisoning (NDP) fell from 9.4 to 4.5 per 100,000 inhabitants, and from 2.5 to 1.6 per 1 million inhab., respectively.

**Implications for services**

The findings indicate the need to identify the risk factors, causes and circumstances of the occurrence of NDP, providing evidence for effective prevention measures.

**Perspectives**

Hospitalizations due to NDP continue to be a serious public health problem, due to its impact on individual and collective health as well as the environmental risks. The preventable nature of NDP highlights the need for new prevention efforts.

## Introduction

With effect from the beginning of the 21^st^ century, there has been a rising trend in the use of chemicals in the global economy and in daily modern life, which may be related to the increase in human exposure to these products.^1^ ^-^ ^3^ According to the World Health Organization (WHO), approximately 2 million lives and 53 million disability-adjusted life years (DALYs) were lost due to exposure to chemicals in 2019.^4^ These estimates are higher than those for 2016 (1.6 million lives and 45 million DALYs) and 2012 (1.3 million lives and 43 million DALYs).^4^ It is important to highlight that the data available on exposure to chemicals is limited to a small number of chemical agents and, therefore, such exposure may also be caused by a wide variety of other substances.

In Brazil, the National Toxicological and Pharmacological Information System (*Sistema Nacional de Informações Tóxico-Farmacológicas* - SINITOX) recorded a total of 97,398 poisoning cases and 445 deaths due to poisoning (0.5%) in 2016.^5^ Among the agents reported, non-drug chemicals were the main cause of poisoning, corresponding to 33.0% of the cases notified in this period. The number of cases of poisoning registered on the SINITOX system has been decreasing in recent years, which is due to the reduced participation of the Centers for Toxicological Information and Assistance (*Centros de Informação e Assistência Toxicológica*) in these surveys, as shown in this database.^5^

Exposure to poisonous substances is a relevant public health problem in a variety of countries,^6^ ^-^ ^8^ considering that the process of industrialization is not always accompanied by knowledge, labor laws and institutional structures for the protection of people and the environment. Data on non-drug poisoning (NDP) in Brazil is scarce and incomplete. As such, the objective of this study was to determine the rate of hospitalizations due to acute NDP events and to analyze mortality arising from NDPs in Brazil, including the trends identified during the ten-year period from 2009 to 2018.

## Methods

This was a time series study, the aim of which was to determine NDP hospitalization and death rates in Brazil. 

We analyzed information on NDP hospitalizations and deaths available on the Ministry of Health Hospital Information System (*Sistema de Informações Hospitalares* - SIH/SUS) database for the period from 2009 to 2018. The data on NDP hospitalizations and deaths held on SIH/SUS were retrieved using the TabWin application. The 2010 Census was used as the source of demographic data on the number of inhabitants used in the rate denominators. For the other years covered by the study we used the intercensal population estimates provided by the Brazilian Institute of Geography and Statistics (*Instituto Brasileiro de Geografia e Estatística* - IBGE).^9^

We initially selected hospitalizations from 2009 to 2018, in which the procedure requested on the Hospital Admission Authorization form (*Autorização para Internação Hospitalar* - AIH) was “treatment of intoxication or poisoning by exposure to drugs and non-drug substances”. Only hospitalizations in which the procedure indicated above was confirmed were kept in the analysis, and we excluded hospitalizations for which this procedure was not confirmed in the final AIH report. In this study, we only analyzed cases of hospitalization due to NDP; those caused by drugs were analyzed separately in a study published elsewhere.^10^

The following variables were included in the study: sex (male; female), age in years or by age group (< 5; 5-9; 10-14; 15-19; 20-29; 30-39; 40-49; 50-59; 60-69; 70 or over), self-reported race/skin color (White; mixed race; Black; other; not informed), place of residence (municipality and region) and hospitalization outcome (discharge/transfer; death).

The hospitalization rate was calculated by dividing the total number of cases of hospitalization due to NDP by the number of inhab. in the respective period and place of residence of the cases. Similarly, the mortality rates were calculated by dividing the total number of deaths due to NDP by the total population in each period studied. These rates were calculated by sex, age and geographical region of residence in Brazil. The hospitalization case fatality ratio was calculated by dividing the number of deaths by the total number of hospitalizations. Relative risk (RR) was estimated as the ratio between rate in a given group compared to the reference group, and the 95% confidence intervals (95%CI) were based on the assumption that the events found have a Poisson distribution.^11^

As crude rates are influenced by the age composition of populations in different regions and in different periods, we used the direct method for standardization of estimated rates by age group, using the standard population suggested by the WHO (WHO, 2000-2025). This enabled rates to be compared and trends to be assessed over the study period.^12^

The time series trend was analyzed using the Prais-Winsten generalized linear regression method, correcting for the effect of first-order autocorrelation. The death/hospitalization trend was considered stationary when the p-value was > 0.05; falling when the p-value was < 0.05 and the regression coefficient was negative; or rising when the p-value was < 0.05 and the regression coefficient was positive.^13^ The statistical analyses were performed using Stata (Stata Statistical Software: Release 16. College Station, TX: StataCorp LLC).

The study was conducted using public domain free access information, whereby data privacy and confidentiality were guaranteed. The study project was therefore exempted from submission to and assessment by a Research Ethics Committee.

## Results

In the period from 2009 to 2018, 276,568 chemical use-related hospitalizations were identified, distributed over 5,351, municipalities in all 26 Brazilian states. Of this total, 125,570 (45.4%) were due to NDP. Average annual incidence of hospitalizations due to NDP was 6.28 per 100,000 inhab. Among the hospitalizations due to NDP, there were 4,326 (3.4%) deaths, corresponding to an average annual mortality rate of 2.16 per 1 million inhab.

Table 1 shows the distribution of the number of NDP hospitalizations and deaths, by geographic region and sociodemographic characteristics of the cases. Most hospitalizations occurred in males (62.1%), the average annual rate (8.0 per 100,000 inhab.) of which was higher than that found for females (4.6 per 100,000 inhab.). Approximately one third of the hospitalization deaths resulting from NDP occurred in females (32.3%).

**Table 1:** Frequency of hospitalizations and deaths due to non-drug poisoning, according to selected characteristics, Brazil, 2009-2018

Variables	Hospitalizations		Deaths	
	n	%	n	%
Total	125,570	100.0	4,326	100.0
**Sex**				
Female	47,477	37.8	1,399	32.3
Male	78,093	62.2	2,927	67.7
**Age group (in years)**				
Under 5	10,809	8.6	53	1.2
5-9	3,575	2.8	18	0.4
10-14	4,566	3.6	66	1.5
15-19	9,789	7.8	235	5.5
20-29	21,656	17.2	632	14.6
30-39	25,364	20.2	870	20.1
40-49	23,968	19.1	972	22.5
50-59	14,884	11.9	690	16.0
60-69	6,564	5.2	430	9.9
70 or over	4,395	3.6	360	8.3
**Race/skin color**				
White	38,684	30.8	1,204	27.8
Mixed race	37,480	29.9	1,235	28.6
Black	4,583	3.6	157	3.6
Other	1,293	1.0	51	1.2
Not informed	43,530	34.7	1679	38.8
**Region**				
North	7,462	5.9	215	5.0
Northeast	30,555	24.3	1,382	31.9
Midwest	13,071	10.4	278	6.4
Southeast	58,017	46.2	2,070	47.9
South	16,465	13.2	381	8.8

The NDP mortality rate was 1.36 per 1 million inhab. for females and 2.96 per 1 million inhab. for males. White race/skin color was most frequently reported with regard to hospitalizations (30.8%); in 34.7% of cases, information on skin color was missing. There were more NDP-related hospitalizations (46.2%) in the Southeast region, followed by the Northeast (24.3%) and the South (13.1%). 

**Figure 1:**
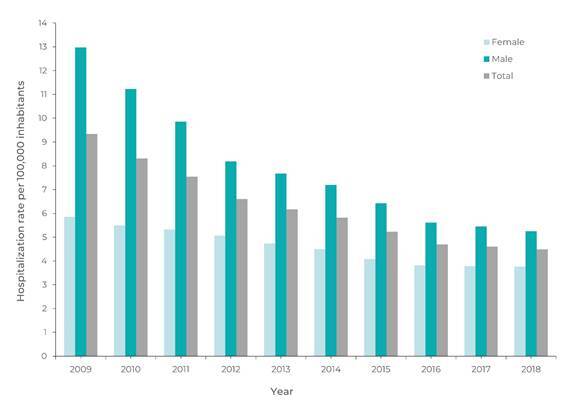
Hospitalization rate due to non-drug poisoning by sex and year, Brazil, 2009 to 2018

The overall NDP hospitalization rate decreased from 9.3 to 4.5 per 100,000 inhab. Figure 1 shows the NDP hospitalization rate by sex and year. The average rate of NDP hospitalizations in males (8.0 per 100,000 inhab.) was higher than that found for females, i.e. 4.6 per 100,000 inhab., RR = 1.73 (95%CI 1.21;2.54). The NDP hospitalization rate decreased in both sexes: in males, it fell from 12.9 to 5.3 per 100,000 inhab.; while in females it fell from 5.9 to 3.8 per 100,000 inhab.

**Figure 2:**
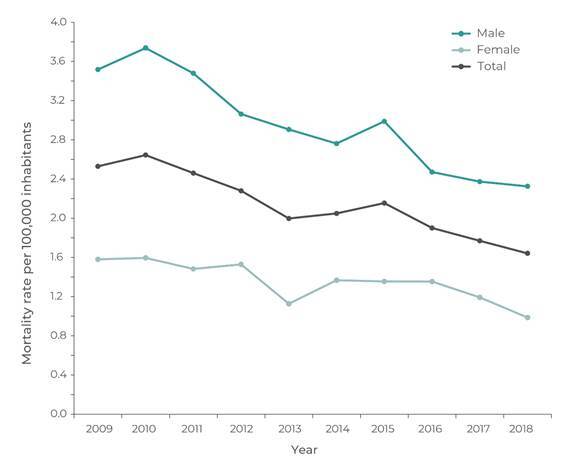
Hospitalization mortality due to non-drug poisoning by sex and year, Brazil, 2009 to 2018

Figure 2 shows NDP hospitalization mortality rates by sex and year. The average NDP hospitalization mortality rate in males (2.96 per 1 million inhab.) was higher than that found in females (1.36 per 1 million inhab., RR = 2.14 (95%CI 1.10;4.03). During the decade analyzed, NDP hospitalization mortality decreased in both sexes, from 3.52 to 2.33 per 1 million inhab. for males, and from 1.58 to 0.99 per 1 million inhab. for females. The overall NDP hospitalization mortality rate decreased from 2.54 to 1.66 per 1 million inhab.

Table 2 shows the NDP hospitalization rate and mortality rate by age. The highest rate of NDP hospitalizations was found in the 30-49 age group, followed by rate in those under 5 years of age. Over the study period, average NDP mortality was 2.16 per 1 million inhab. Individuals aged 40-49 years had the highest mortality rate, followed by those aged 50-59 years. At the beginning of the period studied (2009), the highest hospitalization rate was found in the 30-59 age group, but by the end of the decade, a higher rate was found in children under 5 years old. There was a reduction in NDP hospitalization in general and in all age groups, except for the 15-29 age group, which increased towards the end of the series, and except for the 5-14 age group, which remained stable.

**Table 2:** Hospitalizations and mortality rates due to non-drug poisoning by age group, Brazil, 2009 to 2018

Variables	Year										Annual Average
	2009	2010	2011	2012	2013	2014	2015	2016	2017	2018	
**Hospitalization rate^a^**											
**Age group (in years)**											
< 5	9.1	8.7	8.9	8.5	8.4	8.2	7.8	7.5	7.3	7.1	8.1
5-9	3.0	2.8	2.8	2.8	2.4	2.5	2.2	2.1	2.0	2.0	2.5
10-14	3.2	3.0	2.9	2.9	2.8	2.7	2.7	2.3	2.7	2.7	2.8
15-19	6.5	6.4	6.3	6.1	5.9	5.5	4.7	4.4	5.1	6.1	5.7
20-29	9.9	8.8	8.0	6.8	6.5	6.2	5.6	5.1	5.1	5.4	6.7
30-39	13.7	11.8	9.9	8.7	7.8	7.4	6.6	5.5	5.3	5.2	8.2
40-49	15.2	12.8	11.4	9.8	8.8	7.7	6.8	6.0	5.7	5.1	8.9
50-59	11.3	9.3	8.4	7.2	6.3	6.4	5.5	5.0	4.7	4.2	6.8
60-69	7.2	6.1	5.5	4.5	4.7	4.1	3.9	3.5	3.1	3.0	4.6
70 or over	5.5	5.3	4.9	3.8	3.8	4.0	3.4	3.0	2.6	2.3	3.9
Total	9.4	8.3	7.5	6.6	6.2	5.8	5.2	4.7	4.6	4.5	6.3
**Mortality^b^**											
**Age group (in years)**											
< 5	0.59	0.44	0.59	0.37	0.37	0.61	0.15	0.15	0.54	0.15	0.40
5-9	0.13	0.45	0.13	0.21	0.07	0.00	0.07	0.00	0.15	0.00	0.12
10-14	0.34	0.46	0.57	0.41	0.24	0.43	0.45	0.46	0.33	0.34	0.40
15-19	1.58	1.57	2.08	0.92	1.32	1.44	1.33	0.98	1.06	1.40	1.37
20-29	2.73	2.42	2.66	1.72	1.67	1.67	1.84	1.90	1.42	1.67	1.97
30-39	3.66	3.78	2.52	3.20	2.78	2.70	3.01	2.25	2.20	1.85	2.80
40-49	4.55	4.82	4.06	4.16	3.44	3.28	3.49	2.76	2.79	2.61	3.60
50-59	3.72	3.16	3.41	3.78	3.37	3.42	2.91	2.61	2.52	2.36	3.13
60-69	3.30	4.18	3.40	2.70	2.62	2.34	2.58	3.28	2.66	2.23	2.93
70 or over	2.23	3.65	4.47	3.89	2.32	2.98	3.93	3.08	2.57	1.99	3.11
Total	2.54	2.66	2.48	2.31	2.02	2.07	2.18	1.92	1.79	1.66	2.16

a) Hospitalization rate per 100,000 inhabitants; b) Mortality per 1 million inhabitants.

Table 3 shows the NDP hospitalization rate and mortality rate in Brazil, after standardization according to the country’s geographic regions. In the period studied, the highest average NDP hospitalization rate was found in the Southeast and Midwest regions, while in the Northeast and Northern regions the rates were lower. There was a reduction in NDP hospitalization rates in Brazil as a whole and in all the country’s regions. However, in the Southern and Northern regions the rates stabilized in the second half of the period studied. NDP hospitalization mortality was higher in the Southeast and Northeast regions, while the Northern region had the lowest rate. 

**Table 3:** Rates of hospitalization and mortality due to non-drug poisoning, by region, with regression coefficient and trend, Brazil, 2009 to 2018

Variables	Year										Annual average	Coefficient	95%CI^a^	p-value	Trend
	2009	2010	2011	2012	2013	2014	2015	2016	2017	2018					
**Hospitalization^b^**															
**Region**															
North	5.8	5.2	5.5	5.0	5.6	4.2	3.6	3.1	2.9	3.1	4.4	-0.36	-0.47;-0.24	0.001	Falling
Midwest	13.6	9.9	10.3	9.4	9.2	8.2	7.7	6.8	6.1	6.0	8.7	-0.72	-0.92;-0.52	< 0.001	Falling
Northeast	8.0	8.8	7.5	6.1	5.2	4.9	4.3	3.7	3.6	3.4	5.6	-0.62	-0.77;-0.47	< 0.001	Falling
Southeast	10.7	8.7	8.1	7.2	6.6	6.6	5.8	5.3	5.0	4.9	6.9	-0.58	-0.72;-0.43	0.002	Falling
South	8.5	7.2	5.8	5.3	5.5	4.8	4.9	4.7	5.5	5.3	5.8	-0.29	-0.51;-0.07	0.015	Falling
Brazil	9.4	8.3	7.5	6.6	6.2	5.8	5.2	4.7	4.6	4.5	6.3	-0.54	-0.65;-0.43	< 0.001	Falling
**Mortality^c^**															
**Region**															
North	0.99	1.79	1.70	1.08	1.36	1.16	1.15	1.41	1.17	0.82	1.26	-4.38	-11.71;2.95	0.205	Stationary
Midwest	2.96	2.86	2.81	1.96	1.27	1.84	1.49	0.83	1.07	1.55	1.86	-21.75	-32.67;-10.83	0.002	Falling
Northeast	2.81	2.95	2.55	2.62	2.52	2.49	2.51	2.51	1.82	2.24	2.50	-5.24	-12.01;1.47	0.004	Stationary
Southeast	2.94	2.98	2.78	2.69	2.15	2.35	2.60	2.04	2.30	1.72	2.46	-11.81	-17.36;-6.26	0.001	Falling
South	1.63	1.61	1.78	1.41	1.43	1.08	1.24	1.30	0.95	0.91	1.33	-8.73	-12.27;-5.20	0.003	Falling
Brazil	2.55	2.67	2.48	2.30	2.01	2.07	2.17	1.92	1.78	1.65	2.16	-10.57	-13.42;-7.72	< 0.001	Falling

a) 95%CI: 95% confidence interval; b) Hospitalization rate per 100,000 inhabitants standardized to the distribution of the global population (WHO, 2000-2025); c) Mortality rate per 1 million inhabitants standardized to the distribution of the global population (WHO, 2000-2025).

## Discussion

Cases of NDP hospitalization were reported in all the Brazilian states during the study period (2009-2018). NDPs were the most frequent cause of hospitalization due to poisoning by exposure to drugs and non-drug substances. However, there was a reduction in the NDP hospitalization and mortality rates during the decade analyzed. 

In comparison, another study covering the same period found that hospitalizations due to prescription drugs accounted for 30.1% of hospitalizations, while hospitalizations due to use of over-the-counter drugs were less frequent (0.9%).^10^ Poisoning hospitalizations and mortality roughly halved by the end of the ten-year period from 2009 to 2018. WHO data show a mortality rate of 4.0 per 1 million inhab. in the Americas in 2018.^14^ The estimates are comparable to those produced in a review of poisoning cases in Taiwan for the period 1999-2008, according to which average rate of NDP hospitalizations was 4.97 per 100,000 inhab., while mortality was about 5.5 per 1 million inhab.^15^ However, an upward trend in poisoning mortality and hospitalization rates was found during the period covered by our study (2009-2018). In Finland, a study reviewing two years of hospitalizations (1987-1988) found an NDP hospitalization rate of 30 per 100,000 inhab.,^16^ with rates of chemical poisonings reducing significantly. In another study with children under 5 years old in England between 2000 and 2011, the NDP hospitalization rate decreased, from 41 per 100,000 to 32 per 100,000 inhab.^17^ Incidence of chemical poisoning can vary by geographic region, due to socio-demographic differences and other factors that influence this form of poisoning. Chemical poisoning cases have social, economic and cultural particularities, and may result in distinct patterns between countries and even between regions within the same country.^18^ In addition, mortality attributed to chemical poisoning can also differ according to the age of the victim, administration route and the nature and amount of the chemical, among other factors.^3^ ^,^ ^4^

The age groups most affected by NDP were adults (30-40 years old) and those under 5 years old. In the same period, hospitalizations due to drug poisoning in Brazil also occurred most commonly in children under 5 years old.^10^ In a study conducted in Australia, the rate of hospitalization due to poisoning peaked in the second year of life in the case of non-medical substances.^19^ Accidental intake of medicines or chemicals is most common from 2 to 5 years of age.^20^ Children’s exploratory behaviors and curiosity lead them to touch, test and explore their surroundings, thus coming into contact with toxic chemicals that are not stored safely, especially pharmaceutical and cleaning products.^21^ Furthermore, in this age group lower body weight makes them more vulnerable to poisoning with proportionally smaller amounts of chemicals or medications.^22^ In most cases, chemical poisoning involving children occurs accidentally, and could be avoided if more attention were paid to preventive measures.^21^ ^,^ ^23^ Adults, in turn, are more subject to occupational exposure to chemicals, in addition to intentional poisoning events, which are more frequent in this group than among children.^3^ ^,^ ^8^

Rate of hospitalizations due to NDP poisoning was approximately double in males compared to females. This finding is consistent with WHO data that indicates a higher global rate of mortality due to unintentional poisoning in males.^14^ Analysis of deaths due to poisoning that occurred in Brazil between 2010 and 2015 found that the mortality rates reported for poisoning were higher for the males.^24^ In another review of data on the profile of mortality due to poisoning in Brazil over a ten-year period (from 1996 to 2005), higher frequency of deaths due to drug poisoning was also reported among males.^25^ Differences in male lifestyles and behaviors, as well as occupational exposure to chemicals, which is more frequent among men, are possible reasons for the results found.

The highest mortality rates were found in individuals aged 40 years or older, similarly to what has been found by other Brazilian studies in which mortality due to drug poisoning was analyzed.^26^ ^,^ ^27^ Occupational exposure to chemicals also occurs more frequently in this age group; additionally, with increasing age, there is the possibility of a cumulative effect of exposure to toxic substances. In addition, increased susceptibility to toxicity, associated with decreased capacity for metabolization and excretion of toxins that occurs in people as they get older, may contribute to higher mortality in this age group.^27^

Incidence of poisoning-related hospitalizations varied by region of the country. The highest rate of NDP hospitalization occurred in the Midwest region, while the North and Northeast regions had the lowest hospitalization rates. In the same period, the Midwest region also had the highest rate of drug poisoning hospitalizations in Brazil, while in the North and Northeast regions hospitalizations for this reason were less common.^10^ Similarly, NDP mortality also varied by region of the country, being highest in the Northeast region and lowest in the Northern region. In a review of deaths between 2010 and 2015, poisoning mortality rates were highest in the Northeast and Midwest regions.^24^ Thus, it is possible that the differences found in our study result from differences in the availability of and access to chemicals by people living in different regions of the country. Additionally, it is possible that they are a consequence of discrepancies in the coverage of health services responsible for reporting poisoning cases and deaths in Brazil.^28^

There was a downward trend in the rate of NDP hospitalizations and mortality. These results are consistent with falls in poisoning hospitalization rates reported in Brazil^24^ and in other countries.^17^ ^,^ ^29^ The decrease in cases may be due to the population’s growing awareness about the problem of chemical poisoning, its complications and methods of prevention. Regarding the adult population, the decreasing trend in the number of people employed in the agricultural industry, the increasing importance attributed to environmental protection and occupational safety, by the public in general and by regulatory agencies, are possible reasons for the reduction in the rate of NDP hospitalizations and mortality found in our study.^24^ ^,^ ^28^

Regarding children, probable explanations for the drop seen in poisonings in this group include legislative changes, public awareness, as well as the impact of targeted public health education programs.^30^ The success of public health interventions to prevent childhood poisoning, such as the application of child-proof lids and locks, can reduce the number of poisoning deaths.^21^ However, these devices are not a substitute for safe storage and parental supervision.^23^

The retrospective nature of this study - given that it was based on a pre-existing database, in which the available information is subject to limitations - prevented us from investigating certain characteristics regarding the causes, circumstances, type of chemical substance and treatment of NDP hospitalizations. Moreover, the information is subject to underreporting. In addition, it refers only to public health services and does not include private ones. The resulting rates are therefore underestimated. On the other hand, given the nationwide coverage of the data we used, including a long ten-year time series, it was possible to compare the country’s various regions and to analyze long-term trends in the NDP hospitalization and mortality rates.

NDP poisoning hospitalizations and mortality were most common in males, in the 30-39 age group and in children under 5 years of age; these rates have roughly halved during the ten-year period from 2009 to 2018. Nevertheless, NDP hospitalizations are a serious public health problem because of the impacts on individual and collective health, the high economic and social cost, the risks to the environment, and particularly given that NDP is preventable. The results of this study indicate that further work is needed to investigate the risk factors, causes and circumstances of the occurrence of this form of poisoning, providing evidence to support new prevention efforts 
